# Differences in Demographics, in-Hospital Management and Short-Term Prognosis in Admissions for Acutely Decompensated Heart Failure to Cardiology vs. Internal Medicine Departments: A Prospective Study

**DOI:** 10.3390/jcdd10080315

**Published:** 2023-07-26

**Authors:** Maria-Anna Bazmpani, Christos A. Papanastasiou, Vasilios Giampatzis, Vasileios Kamperidis, Thomas Zegkos, Pantelis Zebekakis, Christos Savopoulos, Haralambos Karvounis, Georgios K. Efthimiadis, Antonios Ziakas, Theodoros D. Karamitsos

**Affiliations:** 1 First Cardiology Department, Aristotle University of Thessaloniki, AHEPA University Hospital, 54636 Thessaloniki, Greece; mariannabaz@hotmail.gr (M.-A.B.); cpapanas@gmail.com (C.A.P.); vkamperidis@outlook.com (V.K.); zegkosth@gmail.com (T.Z.); hkarvounis@hotmail.com (H.K.); geythymi@auth.gr (G.K.E.); aziakas@auth.gr (A.Z.); 2 Cardiology Department, General Hospital of Kavala, 65500 Thessaloniki, Greece; vgiampatzis@yahoo.com; 3 Fisrt Department of Internal Medicine, School of Medicine, Aristotle University of Thessaloniki, AHEPA University Hospital, 54636 Thessaloniki, Greece; pzempeka@auth.gr; 4 First Propedeutic Department of Internal Medicine, School of Medicine, Aristotle University of Thessaloniki, AHEPA University Hospital, 54636 Thessaloniki, Greece; csavvopo@auth.gr

**Keywords:** acute heart failure, internal medicine department, short-term outcome, prognosis, heart failure hospitalizations

## Abstract

Heart failure (HF) is among the leading causes of unplanned hospital admissions worldwide. Patients with HF carry a high burden of comorbidities; hence, they are frequently admitted for non-cardiac conditions and managed in Internal Medicine Departments (IMD). The aim of our study was to investigate differences in demographics, in-hospital management, and short-term outcomes of HF patients admitted to IMD vs. cardiology departments (CD). A prospective cohort study enrolling consecutive patients with acutely decompensated HF either as primary or as secondary diagnosis during the index hospitalization was conducted. Our primary endpoint was a combined endpoint of in-hospital mortality and 30-day rehospitalization for HF. A total of 302 patients participated in the study, with 45% of them admitted to IMD. Patients managed by internists were older with less pronounced HF symptoms on admission. In-hospital mortality was higher for patients admitted to IMD vs. CD (21% vs. 6%, *p* < 0.001). The composite endpoint of in-hospital death and heart failure hospitalizations at 30 days post-discharge was higher for patients admitted to IMD both in univariate [OR: 3.2, 95% CI (1.8–5.7); *p* < 0.001] and in multivariate analysis [OR 3.74, 95% CI (1.72–8.12); *p* = 0.001]. In addition, the HF rehospitalization rate at 6 months after discharge was higher in IMD patients [HR 1.65, 95% CI (1.1, 2.4), *p* = 0.01]. Overall, HF patients admitted to IMD have worse short-term outcomes compared to patients admitted to CD.

## 1. Introduction

Heart failure (HF) constitutes a major public health problem that is not only associated with unfavorable patient outcomes but also imposes a huge economic burden on healthcare systems worldwide [1,2]. Despite the rapidly evolving advances in the medical and interventional field, the morbidity and mortality rates of the disease remain disappointing [3]. Importantly, only a moderate proportion of HF patients receive appropriate guideline—directed treatment, resulting in high readmission rates. Several patient- (i.e., non-compliance) and physician-related factors (i.e., unawareness of HF treatment) have been implicated as potential contributors to this significant “treatment gap” in HF [4]. Cardiologists are primarily responsible for the care of HF patients admitted to hospital. Nonetheless, in most healthcare systems, for various logistic and medical reasons, internists are also involved in the care of such patients [5]. Interestingly, a recent study showed that the prognosis of HF patients is independently associated with the admitting department. Specifically, HF patients admitted to a cardiology department have a lower mortality risk compared to those admitted to other internal medicine departments [6]. Therefore, the aim of our study was to provide an in-depth understanding of the patient profile, in-hospital management, and short-term outcomes of patients with acute HF admitted to cardiology vs. internal medicine departments.

## 2. Materials and Methods

We conducted a prospective, observational cohort study that involved two academic hospitals in Thessaloniki, Greece.

### 2.1. Patient Population

Consecutive patients with an unplanned admission to IMD or CD from November 2018 to July 2022 were included. Patient enrollment was suspended from November 2020 to May 2021 due to urgent measures during the COVID-19 pandemic. Adult patients with acutely decompensated HF of any etiology (either as primary or as secondary diagnosis during the index hospitalization) were considered eligible for the study, irrespective of left ventricular ejection fraction (EF). Following the latest European guidelines, the diagnosis and classification of HF (HF reduced EF, HF mildly reduced EF, HF preserved EF) was based on the presence of relevant signs/symptoms, along with echocardiographic evidence of cardiac dysfunction (systolic and/or diastolic) [7,8]. Patients with congenital heart disease, precapillary pulmonary hypertension of any group, and those initially admitted to the Intensive Care Unit (ICU) or Cardiac Care Unit (CCU) were excluded. Patients crossing over from IMD to CD or vice versa during index hospitalization were also excluded. All participants gave written informed consent. The study was approved by the local ethics committee and was conducted according to principles outlined in the Declaration of Helsinki.

### 2.2. Data Collection

A pre-specified form was used to record important epidemiological and clinical data, such as demographics (age, sex, body mass index), medical history (cardiovascular and non-cardiovascular comorbidities), prior hospitalizations, clinical characteristics, physical examination, echocardiographic findings and therapeutic interventions on admission and during hospitalization. The New York Heart Association (NYHA) class was evaluated on admission by the treating physician. Comorbidities were derived from patients’ medical records. An assessment of laboratory values was performed to establish anemia and chronic kidney disease. Diabetes mellitus was recorded if the patient fulfilled criteria based on laboratory reference values of fasting glucose and hemoglobin A1C or if the patient had been under antidiabetic medications. The presence of lung disease included chronic obstructive pulmonary disease, asthma, pulmonary fibrosis, and established sleep apnoea syndrome requiring continuous positive airway pressure (CPAP). Laboratory parameters (including serum white blood cell count, hemoglobin, serum creatinine, serum potassium, sodium, and C-reactive protein) were recorded as the last value before discharge. B-type natriuretic peptide and troponin-I were recorded on admission. Pharmacological therapy before admission and prescribed medications on discharge were additionally recorded and cross-checked with the national electronic prescription system. The main reason for admission was retrieved from patients’ discharge forms. All participants underwent a comprehensive echocardiographic study in the echocardiography laboratory within 24 h of admission or, in a few cases, in the emergency department.

### 2.3. Endpoints

The primary outcome of our study was the composite of in-hospital mortality and 30-day rehospitalization for HF. In-hospital mortality was recorded as the date of death according to the hospital’s electronic database. HF-related rehospitalization during the 6-month period after discharge was evaluated as a secondary outcome. The time to first HF readmission from discharge was recorded. Serious adverse events, including myocardial infarction, ventricular fibrillation, cardiogenic shock, intubation, cardiac arrest, defibrillation, reperfusion therapy, bradyarrhythmia, device implantation, and renal replacement therapy initiation, were also evaluated during hospitalization. Follow-up strategies, including reevaluation in a HF outpatient clinic, in primary care, or in the absence of any follow-up, were also evaluated. Follow-up information was obtained via phone interviews and by reviewing national health records 30 days and 6 months after the index hospitalization.

### 2.4. Statistical Analysis

Kolmogorov-Smirnov test was used to test the normality of the data. Student’s *t*-test or Mann-Whitney U test (depending on normality test results) compared continuous variables, while chi-squared tests compared categorical variables. Continuous data are presented as mean ± standard deviation or as median with interquartile range (IQR), and categorical variables are presented as absolute numbers and percentages. Univariable and multivariable regression analyses were used to investigate predictors of outcomes. Variables that were statistically significant in univariate analysis were entered into a stepwise logistic regression model to identify independent predictors of the primary endpoint. Results of logistic regression are given as odds ratio (OR) with a corresponding 95% confidence interval (CI), while results of Cox analysis are presented as hazard ratio with the 95% CI. Statistical significance was set to *p* < 0.05 (two-tailed). All data were analyzed by IBM SPSS Statistics version 24.

## 3. Results

### 3.1. Baseline Characteristics

**Figure 1:**
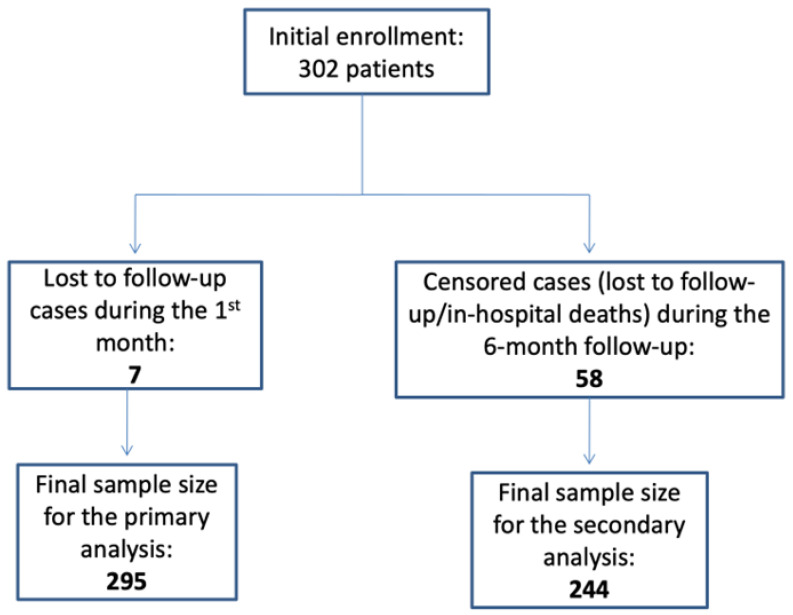
Study population flowchart.

From November 2018 to July 2022, a total of 302 patients with acutely decompensated HF provided informed consent and were enrolled in the study. Censoring of lost to follow-up cases led to a final sample size of 295 patients for the primary analysis and 244 patients for the secondary analysis (Figure 1). The number of patients admitted to IMD was 137 (45%). Acute heart failure (AHF) was the main reason for admission (53%), followed by infections (30%), exacerbation of chronic obstructive pulmonary disease (15%), arrhythmias (4%), ACS (3.3%), while hypertensive emergencies, acute renal failure, electrolyte disorders, and metabolic abnormalities accounted for the rest of admissions in the entire cohort. The majority of patients with AHF had acute decompensation of chronic HF (87%), and only 13% presented with de novo HF. Seven patients (4.2%) presented with acute pulmonary edema, and all of them were admitted to CD. The vast majority of patients with heart failure precipitated by infection were treated by Internal Medicine doctors (62.8% vs. 4.2%, *p* < 0.001). The baseline characteristics of our cohort are summarized in Table 1. In comparison to patients admitted in CD, patients admitted to IMD were older [median age 81 (38–94) vs. 76 (44–90), *p* < 0.001]. Admissions for HF prior to index hospitalization, as well as the prevalence of important comorbidities (i.e., coronary artery disease, diabetes mellitus, atrial fibrillation, and chronic kidney disease) and smoking habits did not differ significantly between the comparison groups. In contrast, lung disease was more prevalent in patients admitted to IMD (44.5% vs. 20%, *p* < 0.001). Patients hospitalized in CD had more pronounced symptoms of congestive heart failure at presentation, as demonstrated by a higher likelihood of functional NYHA class III or IV (87% vs. 75%, *p* = 0.01) and more specific signs of HF such as orthopnea and paroxysmal nocturnal dyspnea. However, from laboratory investigations, we found no difference in NT-proBNP values between CD and IMD (median values 4550 vs. 4603, *p* = 0.87). Furthermore, patients treated by Cardiologists had significantly lower levels of white blood cells and c-reactive protein and higher levels of hemoglobin and potassium, as shown in Table 1. Finally, left ventricular systolic function (LVEF) was consistently lower in patients admitted to CD compared to that of patients admitted to IMD.

### 3.2. In Hospital Management, in Hospital Events, Discharge Medications and Follow up Strategies

The duration of hospital stay did not differ significantly between the IMD and the CD patients [8 (2–43) vs. 8 (2–63) days, *p* = 0.64)]. In-hospital death occurred more frequently (21% vs. 6%, *p* < 0.001) in patients admitted to IMD. Escalation of HF management with the initiation of inotropes, as well as step-up admission to the intensive care unit (ICU) or cardiac care unit (CCU), were more common in CD patients (15% vs. 5%, *p* = 0.02 and 33% vs. 14.5%, *p* < 0.001; respectively). The occurrence of serious adverse events did not differ significantly between the two groups of patients. However, there was a statistically significant difference in follow-up plans with a higher percentage of CD patients followed up by HF clinics compared to IMD patients (29% vs. 3.7%, *p* < 0.001), as shown in Table 2. Overall, no difference was found in prehospital medications between the IMD and CD patients. A higher prescription rate for angiotensin receptor blockers and calcium channel blockers was noted at discharge for patients treated by internists. A summary of prehospital and discharge medications is presented in Table 3.

### 3.3. Clinical Outcomes

**Figure 2:**
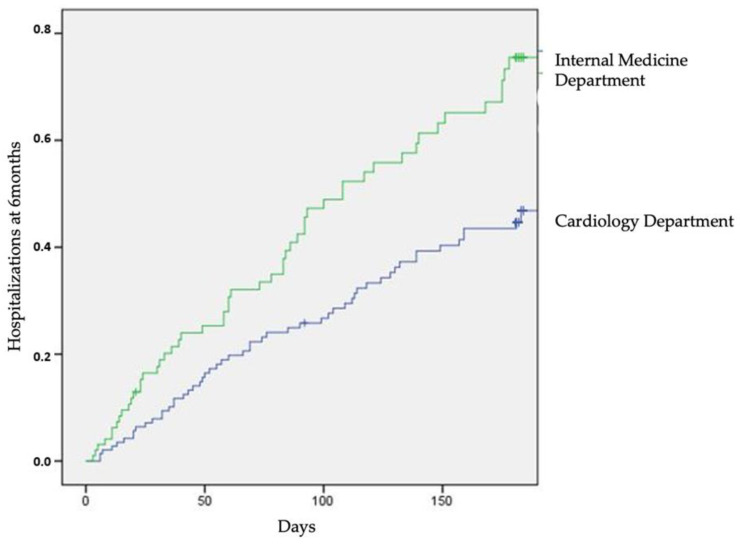
Survival curves for 6-month follow-up for heart failure hospitalizations (6-month readmission rate for HF was significantly different between the two groups (*p* = 0.01).

Admission to IMD was associated with a higher risk for the composite endpoint of in-hospital death and 1-month readmissions for heart failure both in univariate [OR: 3.2, 95% CI (1.8–5.7); *p* < 0.001] and multivariate analysis [OR: 3.74, 95% CI (1.72–8.12); *p* = 0.001]. The results of multivariate analysis are presented in Table 4. Apart from admission to IMD, advanced NYHA (III and IV) was associated with a higher risk for the primary outcome [OR 1.87, 95% CI (1.06–3.3), *p* = 0.03] while a higher left ventricular ejection fraction was associated with a lower risk [OR 0.68, 95% (0.47–0.97), *p* = 0.04]. Finally, based on the results of the secondary analysis, we found that the HF-related readmission rate was higher in IMD patients during the first 6 months after discharge [HR 1.65, 95% CI (1.1, 2.4), *p* = 0.01; Figure 2].

## 4. Discussion

This was a real-world, prospective study that compared patients with acute heart failure admitted to CDs vs. IMDs. The main findings of our study can be summarized in the following key points: (i) Patients admitted to IMDs were more likely to be older, have less pronounced signs and symptoms of HF, and have a higher LVEF. However, no significant difference was found between the two comparison groups in terms of important comorbidities such as coronary artery disease, diabetes mellitus, atrial fibrillation, and chronic kidney disease; (ii) Admitting department was found to be an independent predictor of the combined endpoint of in-hospital mortality and 30-day rehospitalization for HF (OR 3.74 for IMD patients). These findings have important clinical implications since acute decompensated HF is associated with poor prognosis and high readmission rates.

The results of our study are in agreement with those of previous studies [9,10,11]. Reis et al., who studied 298 patients with HF, found that the 6-month readmission rates were higher for patients treated by generalists, while patients treated by cardiologists had longer in-hospital stay, underwent more cardiac diagnostic tests, and were more frequently treated with inotropes [12]. Likewise, in our study, patients treated by cardiologists were more frequently admitted to intensive care or cardiac care units and needed intravenous inotropes. These findings could be partially explained by the fact that patients with advanced HF (i.e., severely impaired left ventricular function) are usually admitted to cardiology departments, as shown in our study. Taking this hypothesis into account, someone would expect that mortality and readmission rates would be higher in patients admitted to cardiology departments. Instead, we found that admission to an internal medicine department is associated with worse short-term outcomes, even after adjusting for important confounders (e.g., age, infection). Similar findings were also reported in a large study by Selim et al., which demonstrated that for patients treated by cardiologists compared to those treated by internal medicine physicians alone, HF readmissions and all-cause mortality at 30 days were significantly lower in patients treated by cardiologists (HR: 0.76, 95% CI: 0.66–0.89, *p* = 0.002) [13]. Increased familiarity of cardiologists with guideline-directed medical therapies and fluid status assessment are possible parameters contributing to better outcomes for patients admitted to cardiology departments. Moreover, it could be hypothesized that cardiologists have a better understanding of the appropriate timing for intensification of treatment in AHF compared to internists, thus providing a more personalized approach in the management of such patients. Additionally, as shown in our study, patients receiving care by cardiologists, are more likely to be referred to dedicated HF clinics with specialist HF cardiologists and nurses and have a stricter follow-up plan including optimization of medical therapies and referral for device therapies [14].

Another fact that deserves attention in our study is the low prescription rates of guideline-directed medical therapies for both patient categories, taking into consideration that the patients in our cohort represent the whole spectrum of ejection fraction. Contrary to our study, where no difference was found in prescribed medications between the CD and IMD patients, previous studies demonstrated that patients treated in a cardiology setting received more common treatment with b-blockers and renin-angiotensin system inhibitors [10,15]. Based on our findings, it seems that internists were more likely to prescribe angiotensin receptor blockers and calcium channel blockers instead of angiotensin-converting enzyme inhibitors. Regarding sodium-glucose cotransporter two inhibitors, there was a trend towards higher prescription rates by Internal medicine doctors at discharge, which could be attributed to the fact that until recently, prescription of SGLT2i was restricted to Internists in our country.

Some limitations should be acknowledged for our study. This study involved two academic institutions; hence, our findings should be extrapolated with caution. As an observational study, it is limited by the inherent constraints of this study design. Because enrollment took place during the COVID-19 pandemic, patients with heart failure and SARS-CoV-2 infection were not included, introducing selection bias. Furthermore, in patients admitted to IMD, cardiology consultation may have been sought and not captured in our analysis. Finally, follow-up details were obtained via telephone, and thus, some readmissions may not have been estimated. However, only seven patients were lost during the follow-up period.

## 5. Conclusions

Our analysis demonstrated that patients with HF admitted to IMD experienced higher In-hospital mortality and more frequent HF readmissions during the early post-discharge period (30 days) and for 6 months. Taking into consideration that heart failure patients admitted to hospital are often managed in a non-cardiology setting and that treating heart failure patients is challenging throughout the trajectory of a hospitalization, additional measures (i.e., multidisciplinary approach involving both cardiologists and internal medicine physicians, early post-discharge reassessment by HF clinics for intensification of guideline-directed medical treatments) should be implemented to improve outcomes of patients hospitalized in internal medicine departments.

## Figures and Tables

**Table 1:** Baseline characteristics of patients with heart failure admitted to Cardiology and Internal Medicine Departments.

	Internal Medicine Patients N = 137	Cardiology Patients N = 165	*p*-Value
Demographics			
Age, years	81 (38–94)	76 (44–90)	**<0.001**
Male sex	68 (49.6%)	86 (52.1%)	0.66
BMI, kg/m^2^	28.4 (20.2–44.9)	28.2 (20–46)	0.56
Smoking			0.59
Never	67 (48.9%)	81 (49.1%)	
Current	48 (35%)	51 (30.9%)	
Former	22 (16.1%)	33 (20%)	
HFH in last 12 months			0.16
Yes	84 (50.9%)	81 (59.1%)	
No	81 (49.1%)	56 (40.9%)	
Primary reason for admission			**<0.001**
Acutely decompensated HF	29 (21.1%)	125 (75.8%)	
Pulmonary edema	0 (0%)	7 (4.2%)	
ACS	0 (0%)	10 (6.1%)	
Arrythmia	0 (0%)	12 (7.3%)	
Acute renal failure	3 (2.2%)	0 (0%)	
COPD exacerbation	14 (10.2%)	1 (0.6%)	
Comorbidities			
CAD	61 (44.5%)	87 (47.3%)	0.63
DM	64 (46.7%)	72 (43/6%)	0.45
AF	99 (72.3%)	111 (67.3%)	0.31
CKD	64 (46.7%)	81 (49.1%)	0.46
Hypertension	96 (70.1%)	105 (63.6%)	0.48
Lung disease	61 (44.5%)	33 (20%)	**<0.001**
Anemia	86 (62.8%)	98 (59.4%)	0.55
Dyslipidemia	75 (54.7%)	90 (54.5%)	0.97
Presenting symptoms			
Dyspnea	126 (92%)	159 (96.4%)	0.10
PND	21 (15.3%)	52 (31.5%)	**0.003**
Fatigue	108 (78.8%)	125 (75.8%)	0.41
Nocturnal cough	41 (29.9%)	21 (12.7%)	**<0.001**
Orthopnea	45 (32.8%)	97 (58.8%)	**<0.001**
Ankle swelling	65 (39.4%)	100 (60.6%)	0.25
Chest pain	24 (17.5%)	26 (15.8%)	0.68
Wheezing	27 (19.7%)	10 (6.1%)	**<0.001**
Palpitations	23 (16.8%)	33 (20%)	0.50
Syncope	10 (7.3%)	6 (3.6%)	0.16
Advanced NYHA (III, IV)	103 (75%)	143 (87%)	**0.01**
Laboratory findings			
Hgb, mg/dL	11.24 (±2.0)	11.4 (±2.0)	**0.007**
NTproBNP(pg/ml)	4603 (451–39,127)	4550 (650–70,000)	0.87
Cr, mg/dL	1.19 (0.37–5.04)	1.36 (0.43–6.17)	0.18
Potassium, mmol/L	4 (2.8–5.6)	4.21 (±0.55)	**0.024**
WBCs	11.250 (3.200–27.000)	7610 (3.000–24.870)	**<0.001**
CRP, mg/dL	5 (0.2–40)	1.16 (0.15–13.3)	**<0.001**
HF classification			**0.04**
HFrEF	32/91 (35.2%)	59/91 (64.8%)	
HFmrEF	28/65 (43.1%)	37/65 (56.9%)	
HFpEF	75/144 (52.1%)	69/144 (47.9%)	

Categorical variables are presented as absolute numbers and percentages. Continuous variables are presented as mean ± standard deviation or as median with interquartile range (IQR). BMI, body mass index; HFH, heart failure hospitalizations; HF, heart failure; CAD, coronary artery disease; DM, diabetes mellitus; AF, atrial fibrillation; CKD, chronic kidney disease; PND, paroxysmal nocturnal dyspnoea; NYHA, New York Heart Association, Hgb, hemoglobin; NT-proBNP, B-type natriuretic peptide; Cr, creatinine; WBCs, white blood cells; CRP, C-reactive protein, HFrEF, heart failure with reduced ejection fraction; HFmrEF, heart failure with mildly reduced ejection fraction; HFpEF, heart failure with preserved ejection fraction. Significant *p*-values are shown in bold.

**Table 2:** In-hospital events and follow-up strategies of patients admitted to IMD and CD.

	Internal Medicine Patients N = 137	Cardiology Patients N = 165	*p*-Value
Length of stay, days	8 (2,43)	8 (2,63)	0.64
In-hospital death, %	29 (21%)	10 (6%)	**<0.001**
Any SAE, %	21 (15%)	23 (14%)	0.25
Need for ICU/CCU	20 (14.5%)	55 (33%)	**<0.001**
Use of inotropes, %	8 (5%)	24 (15%)	**0.02**
Need of iv diuretics, %	129 (94%)	154 (93%)	0.65
Need of iv vasodilators, %	13 (9%)	13 (8%)	0.61
Follow up strategies, %	N = 108	N = 155	**<0.001**
HF clinic	4 (3.7%)	45 (29%)	
Primary care	31 (28.7%)	46 (29.7%)	
None	73 (67.6%)	64 (41.3%)	

Categorical variables are presented as absolute numbers and percentages. SAE, serious adverse event (SAEs included MI, malignant arrhythmias, cardiogenic shock, intubation, device implantation, and initiation of hemodialysis during index hospitalization). Significant *p*-values are shown in bold.

**Table 3:** Prehospital and discharge medications of patients admitted with HF to IMD and CD.

	Internal Medicine Patients	Cardiology Patients	*p*-Value
**Prehospital**	n = 137	n = 165	
ACEi	21 (15.3%)	34 (20.6%)	0.13
ARB	47 (34.3%)	33 (20%)	**0.01**
ARNI	3 (2.2%)	6 (3.6%)	0.42
b-blockers	107 (78.1%)	128 (77.6%)	0.67
MRA	44 (32.1%)	72 (43.6%)	0.12
SGLT2i	10 (7.3%)	7 (4.2%)	0.31
Loop diuretics	104 (75.9%)	128 (77.5%)	0.79
CCBs	52 (38%)	44 (26.7%)	0.11
Statins	62 (45.2%)	81 (49%)	0.31
**Discharge**	n = 108	n = 155	
ACEi	23 (21.3%)	33 (21.3%)	0.70
ARB	27 (25%)	17 (11%)	**0.008**
ARNI	4 (3.7%)	17 (11%)	0.08
b-blockers	94 (87%)	135 (87.1%)	0.20
MRAs	54 (50%)	97 (62.6%)	0.13
SGLT2i	31 (28.7%)	27 (17.4%)	0.07
Loop diuretics	104 (96.3%)	144 (92.9%)	0.43
CCBs	37 (34.3%)	31 (20%)	**0.02**
Statins	52 (48.1%)	85 (54.8%)	0.25

Categorical variables are presented as absolute numbers and percentages. ACEi, angiotensin-converting enzyme inhibitors; ARB, angiotensin II receptor blockers; ARNI, angiotensin receptor blocker, and neprilysin inhibitor; MRA, mineralocorticoid receptor antagonist; SGLT2i, sodium-glucose cotransporter-2 inhibitors; CCBs, calcium channel blockers. Significant *p*-values are shown in bold.

**Table 4:** Predictors of the composite endpoint of in-hospital mortality and 1-month readmissions (Multivariate analysis including only significant variables).

Variable	OR	95% CI	*p*-Value
Age	0.97	0.97–1.03	0.72
Admission to IMD	3.74	1.72–8.12	**0.001**
Advanced HYHA class (III, IV)	1.87	1.06–3.30	**0.03**
LVEF	0.68	0.47–0.97	**0.04**
Lung disease	1.61	0.81–3.19	0.17
CRP	1.03	0.98–1.08	0.23

OR, odds ratio; CI, confidence interval; IMD, Internal medicine department; LVEF, left ventricular ejection fraction; CRP, c-reactive protein. Significant *p*-values are shown in bold.

## Data Availability

The data presented in this study are available on request from the corresponding author. The data are not publicly available due to privacy and ethical restrictions.
